# SUCCEED Africa: protocol for a multi-method pilot study of a community-based intervention for people with psychosis in Sierra Leone, Nigeria, Zimbabwe and Malawi

**DOI:** 10.1186/s40814-024-01536-x

**Published:** 2024-08-27

**Authors:** Rachel Greenley, Rita Tamambang, Alhaji Koroma, Bisola Fasoranti, Ephiphania Munetsi, Hilda Chinoko, Nancy Stevens, Nyaradzo Goba, Philani Ama Kinyabo, Tolulope Bella-Awusah, Grace Ryan

**Affiliations:** 1 Centre for Global Mental Health, London School of Hygiene and Tropical Medicine, Keppel Street, London, WC1E 7HT UK; 2 Centre for Child and Adolescent Mental Health, College of Medicine, University of Ibadan, Ibadan, Nigeria; 3 Department of Child and Adolescent Psychiatry, University College Hospital, Ibadan, Nigeria; 4 Department of Psychiatry, College of Medicine, University of Ibadan, Ibadan, Nigeria; 5 Mental Health Department, University of Makeni, Lunsar-Makeni Highway, Makeni, Sierra Leone; 6 Research Support Centre, Faculty of Medicine and Health Sciences, University of Zimbabwe, Avondale, Harare, Zimbabwe; 7 Department of Mental Health, Kamuzu University of Health Sciences, P/Bag 360, Blantyre, Malawi

**Keywords:** Psychosis, Psychosocial disabilities, Co-production, Peer support, Community-based rehabilitation, Community mental health, Sub-Saharan Africa

## Abstract

**Background:**

Recent reviews have highlighted the need for participatory research to design and evaluate inclusive, community-based interventions that address the diverse needs of people with lived experience of psychosis, within and beyond the health sector. The SUCCEED Africa consortium aims to co-produce a 6-year programme of research across four countries in West (Sierra Leone, Nigeria) and Southeast Africa (Zimbabwe and Malawi). This protocol describes the pilot study in which SUCCEED’s intervention, research tools and processes will be tested on a small scale in each country in preparation for future evaluation research.

**Methods:**

The SUCCEED intervention comprises peer support, case management and livelihood activities for people with lived experience of psychosis. The pilot uses a before-and-after study design investigating change in subjective quality of life in adults diagnosed with a primary psychotic disorder or another mental disorder with psychotic symptoms who are offered the SUCCEED intervention over a 4-month period. Nested within this study are the following: a baseline assessment of the feasibility, acceptability and face validity of the selected measurement tool and validity of proxy versus self-completion; and a multi-method process evaluation examining key process indicators and implementation, service and client-level outcomes. Methods include the following: baseline cognitive interviews; semi-structed observation and routine monitoring and evaluation of service delivery; endline interviews and focus group discussions; and a comparison of provider competencies at endline. At each of the four pilot sites, participants will include the following: ten people with lived experience of psychosis, recruited from either health services or community settings using purposive sampling to maximise variation; up to ten adult family members (one per participant with lived experience) involved in their care; the peer support worker, community support worker and supervisor responsible for delivering the intervention; and the data collectors. Recruitment will take place in July and August 2023.

**Discussion:**

To the best of our knowledge, this will be the first study of a community-based intervention incorporating lay-delivered case management, formal peer support and livelihoods activities for people with lived experience of psychosis in sub-Saharan Africa. Findings will be relevant not only to SUCCEED but also to others interested in promoting rights-based approaches to community mental health in low-resource settings.

**Trial registration:**

US National Library of Medicine (ClinicalTrials.gov), Protocol reference ID 28346. Initially registered retrospectively July 20/2023: In review.

**Supplementary Information:**

The online version contains supplementary material available at 10.1186/s40814-024-01536-x.

## Background

### Psychoses and disability in sub-Saharan Africa

Mental, neurological and substance use disorders are among the top five contributors to the global disease burden and are the leading cause of years lived with disability (YLDs) worldwide [1]. Although psychotic disorders like schizophrenia and schizoaffective disorder typically affect around 1% of the population [2], they contribute substantially to global disability [3–5]. These disorders are characterised by psychotic symptoms^1^ (e.g. delusions, hallucinations, disorganised thinking) that can be profoundly disturbing and negatively impact daily living [1]. However, the social and economic consequences of psychotic disorders can be just as troubling as the symptoms themselves. Largely due to stigma and discrimination, people with psychotic disorders (and in many cases, their family members) have a high risk of experiencing abuse [6], extreme poverty [7], homelessness [8], incarceration [9], unemployment and reduced educational attainment [10], among other adverse outcomes[11–13].

In many low- and middle-income countries (LMICs), including in sub-Saharan Africa [6, 14], 80–90% of people with psychotic disorders do not receive treatment [3]. This is in part due to weak mental health systems as well as stigma and discrimination, compounded by social and cultural factors that affect help-seeking [15, 16]. Most of the treatment that is available in this region is delivered at specialist hospitals, where forced restraint, prolonged seclusion and other forms of coercive treatment have been reported [4–8]. For example, a recent study of two psychiatric hospitals in KwaZulu-Natal found that more than 70% of involuntarily admitted patients (most of the hospitals’ inpatients) experienced high levels of coercion and threats at the time of admission [17]. Sexual assault and other abuse in family homes, health facilities, social care institutions, traditional and spiritual healing centres and other community settings have also been documented [4–6, 9]. The availability of non-pharmacological interventions is extremely limited [5, 18, 19], despite recognition that antipsychotic medication alone is insufficient to address the complex social, economic and health needs of people with psychotic disorders [20].

A recent systematic review identified 10 studies evaluating the impact of interventions for people with psychotic disorders in Africa, excluding pharmacological trials [5]. Eight of these studies reported positive results across a range of different outcomes (e.g. psychiatric symptoms, substance use, service utilisation, disability, stigma and discrimination, chaining and restraint, caregiver burden), and most used task-sharing approaches to deliver multicomponent interventions. However, these components were overwhelmingly clinical in nature: eight of the included studies involved psychoeducation (for five, this was the only component related to education, awareness or social support), and six involved some form of clinical monitoring, medication prescription or adherence, or appointment reminders. By contrast, only three studies had an empowerment component (self-help groups), two had a livelihoods component (income-generating activities), two had a social component (involving families) and none supported participants’ education. The reviewers concluded that there was a need for further research involving people with lived experience of psychosis in designing and evaluating holistic interventions that meet their diverse needs, within and beyond the health sector.

### Community-based rehabilitation for psychosocial disabilities

Recognising the need for a framework for community-based interventions supporting people with psychosocial disabilities in LMICs, the World Health Organisation’s (WHO’s) 2010 Community-Based Rehabilitation (CBR) Guidelines included a special supplement on mental health which drew mainly from expert opinion, evidence in community mental health, and basic development principles to make recommendations for best practice [21]. However, a 2016 review examining CBR for psychosocial disabilities in LMICs identified only one study from sub-Saharan Africa (South Africa) [18, 19]. The authors concluded that there was a need for more evidence from this region in particular. A systematic review of the grey literature on CBR for psychosocial disabilities in LMICs was subsequently undertaken, in case there was in fact evidence available that had been excluded as a result of the previous review’s strict eligibility criteria [22]. The grey literature review identified 33 different CBR programmes, 11 of which were based in sub-Saharan Africa, but the quality of the evidence was generally very poor and too heterogeneous for any sort of meta-analysis. In contrast to other reviews, the grey literature review found that the majority of programmes were not focused primarily on health. Just over half (17) were classified mainly as livelihoods programmes, 11 as empowerment programmes, eight as social programmes, seven as health programmes and five as education programmes.

Systematic differences between the CBR programmes evaluated through controlled studies and those documented in the grey literature suggest a disconnect between how CBR programmes are designed and evaluated for research purposes, versus real-world practice. Most likely, this reflects the priorities and interests of researchers (typically, academic clinicians) interested in maximising the effects of interventions on clinical outcomes such as symptom reduction—as opposed to, for example, personal recovery [23]. SUCCEED Africa seeks to close this gap, by involving people with lived experience, their families and other key stakeholders in their communities, in designing, implementing and evaluating a CBR-based intervention that takes a social (as opposed to a biomedical) perspective on psychosocial disability.

### SUCCEED Africa

SUCCEED Africa is a five-country Health Research Programme Consortium (RPC) with partners in Malawi, Nigeria, Sierra Leone and Zimbabwe as well as a UK-based coordinating centre. The RPC aims to co-produce a 6-year programme of research following a Theory of Change [4]-driven approach aligned with the UK Medical Research Council (MRC) framework for the development and evaluation of complex interventions (see Additional file #1 for a working draft of the ToC map) [24]. This is a common approach to intervention development in global mental health that has previously been used to design a CBR intervention for people with schizophrenia in Ethiopia [21, 25, 26]. Used in concert with the MRC framework, ToC is an iterative process that emphasises the importance of stakeholder engagement and real-world experience in developing, testing and refining a complex intervention [27]. ToC is also increasingly recognised as a useful tool for involving people with lived experience in mental health research in low-resource settings; for example, in Uganda, Ethiopia and Colombia [28–30].

A key output of SUCCEED’s formative research (results forthcoming) is the development and manualisation of an evidence-based intervention for people with lived experience of psychosis in sub-Saharan Africa. The SUCCEED intervention takes the WHO’s CBR Matrix as a point of departure to consider the multifaceted needs of people living with psychosis and other psychosocial disabilities, and how best to meet these needs by mobilising the resources of individuals and families affected, as well as their broader communities. Key features of the SUCCEED Africa intervention include community-based case management delivered by a lay community support worker (CSW) and formal peer support delivered by a person with lived experience of psychosis (“peer support worker”, PSW) as well as a group livelihoods component following an Asset Based Community Development (ABCD) approach [31] (see “Intervention”). This is a strengths-based approach building on existing skills and resources across relevant domains of the CBR matrix, and integrating recovery-oriented components (i.e. formal peer support) in which SUCCEED members have unique expertise [30, 32–34].

The main aims of SUCCEED Africa’s pilot stage are two-fold and are detailed alongside research objectives in Table 1, below.

**Table 1:** Summary of SUCCEED Africa pilot aims and objectives

Aims	Objectives
1. To further test assumptions underlying the Theory of Change	1.1. To deliver the intervention on a small scale
	1.2. To collect data on key process indicators and outcomes (implementation outcomes, service outcomes and client outcomes)
2. To prepare for future implementation research and evaluation of effectiveness	2.1. To inform the development of structured tools for implementation research
	2.2. To assess the acceptability, feasibility, suitability and utility of WHOQOL-BREF as primary outcome measure for trial

### Lived experience involvement

Previous reviews have highlighted the lack of involvement of people with lived experience of mental health conditions in mental health research in LMICs [22, 23, 30], and this is especially the case for people with psychoses. SUCCEED Africa aims to address this by working with people with lived experience of psychosis at all stages of research and ensuring representation in SUCCEED Africa’s oversight structures, including Local and Consortium Advisory Committees and a dedicated Lived Experience Advisory Panel (LEAP). Each country research team includes at least one person with lived experience of psychosis serving as a peer researcher. Peer researchers have been involved in the design and development of this research project and protocol, and will also be involved in data collection, analysis, writing up and dissemination.

## Methods

### Study design

The pilot will use a before-and-after study design without a control group to assess changes in subjective quality of life among participants with lived experience of psychosis who are offered the SUCCEED Africa intervention over a 4-month follow-up period. Nested within this study are several other components:

- A baseline assessment of the feasibility and acceptability of the WHO’s Quality of Life Brief Version (WHOQOL-BREF) as a measurement tool (time taken to administer, proportion of participants who complete the tool, perspectives of data collectors assessed through interviews), face validity (assessed through cognitive interviewing), and the validity of proxy (completed by a close family member) versus self-completion.
- An endline qualitative study using a combination of focus groups (participants with psychosis, family members) and interviews (PSW, CSW, supervisors, data collectors) to further examine the acceptability and feasibility of SUCCEED’s research tools (including WHOQOL-BREF) and processes.
- A process evaluation drawing on semi-structured observations and routine monitoring and evaluation (M&E) of intervention delivery, competency assessment of the two frontline providers (PSW and CSW), research administrative data (adverse events, drop-outs) and endline qualitative data (see above) to assess key process indicators and client-level (satisfaction), service-level (efficiency, safety, effectiveness, equity, patient-centredness, timeliness) and implementation (acceptability, appropriateness, feasibility) outcomes.

We followed Proctor et al.’s (2011) [35] conceptual framework for outcomes of implementation research to structure our investigation of client-level, service-level and implementation outcomes. This protocol was written in adherence to the Standard Protocol Items: Recommendations for Interventional Trials (SPIRIT) and Template for Intervention Description and Replication (TIDieR) checklists [36, 37] (see Additional File #2), approved by ethics committees and other institutional review boards in each of the five SUCCEED partner countries (see “Ethics”) and registered retrospectively on ClinicalTrials.gov (reference number 28346).

### Setting

Each of the four SUCCEED implementing countries has selected a pilot area in collaboration with the local delivery partners (non-governmental or civil society organisations) identified to host the intervention (Table 2). These organisations have a proven track record of providing servicing in the pilot study districts and a long-standing interest in the rights and welfare of people with disabilities, including mental health conditions. In each country, the pilot covers a contiguous area that is sufficiently populated to enable recruitment, but small enough to avoid long distances for participants to travel to group activities. Study sites were also chosen with the catchment areas of future large-scale evaluation research in mind. Further information on SUCCEED sites can be found in the forthcoming cross-country situation analysis by Omobowale and Greenley (in press) [38].

**Table 2:** Key features of pilot site in each SUCCEED country

Country	Description
Malawi	The pilot will be carried out in Mulanje District in Traditional Authority Mabuka. This site is within the catchment area of the Mental Health Users and Carers Association (MEHUCA), the local organisation identified to host the SUCCEED intervention. Mulanje District is located in the Southern Region of Malawi bordering Mozambique and covers an area of 2056 km^2^, with a population of 428,322. It is mainly occupied by the Lhomwe people, but there are also other tribes such as Mang’anja. Chichewa is the most commonly spoken language. Mulanje is also known for its tea-growing industry and for Mount Mulanje, one of the highest peaks in Southern Africa. Mental health services are provided through Mulanje District Hospital, a secondary-level facility that provides outpatient and in-service patient care. The district also has primary care facilities offering non-specialist care for people with mental health conditions based on WHO’s mental health Gap Action Programme (mhGAP). A few local NGOs also provide care and support services to people living with psychosocial disabilities
Nigeria	The pilot will be carried out in the Ibadan North Local Government Area. This is a primarily urban area located within the Ibadan Metropolis, the capital of Oyo state. Ibadan North covers an area of 22 km^2^, with an estimated population of 308,119. The main languages spoken are Pidgin, English and Yoruba. Yoruba is the majority ethnic group, with Hausa, Ibo and other tribes also present. The local economy is mainly dependent on trading, farming, artisanship and civil service. The local government area is home to the University College Hospital, Ibadan, the foremost tertiary hospital in Nigeria. The hospital has a Department of Psychiatry with 64 beds divided across male and female wards, as well as an eight-bed child and adolescent ward. The department also runs a child and adolescent clinic, an outpatient clinic and a consultation-liaison service
Sierra Leone	The SUCCEED pilot intervention will be carried out in the Aberdeen, Lumley and Looting Town communities of Freetown (Western Area Urban District), the capital city of Sierra Leone with an estimated population of 1,500,234 covering an area of 81.48 km^2^. The main languages spoken are Krio (spoken by the Creole), English and Themne, though Creole is the majority ethnic group. The local economy and city council is mainly dependent on trading, transportation, tourism and civil service. The city hosts the Sierra Leone Re-Correctional Prison, the Freetown Psychiatric Hospital and various mental health organisations offering services such as screening, counselling, referrals and provision of medication. Many local government hospitals also have departments of counselling staffed by mental health nurses
Zimbabwe	The pilot will be carried out in Chitungwiza, an urban centre and town of Harare province. The population is 372,000 and the area spans approximately 50 km^2^. The main language is Shona, and most inhabitants belong to the Shona ethnic group. The spoken language is categorised into dialects, and the main dialect in Chitungwiza is Zezuru. The local economy is largely dependent on informal trading and business, with several shopping centres. Chitungwiza has two main hospitals and four polyclinics. At the main hospital to be involved in referrals for the pilot, Chitungwiza Central Hospital, mental health services are offered at the outpatient department, with admissions referred to Harare Psychiatric Hospital. Chitungwiza Hospital has two student counselling psychologists, two social workers and four mental health nurses in post

### Eligibility criteria

All participants must be consenting (or assenting, with guardian consent) adults (age 18 +) at the time of the study. Where possible, age will be confirmed by a form of identity such as national identity card, birth certificate or a driver’s license. Participants must be able to speak either English or one of the other main local languages in each country, namely Chichewa for Malawi, Yoruba for Nigeria, Krio for Sierra Leone and Shona for Zimbabwe. People with lived experience of psychosis and their family members must live within the pilot study area, for logistical purposes. Providers (PSW, CSW) and supervisors must be existing or new staff (hired for the purposes of SUCCEED) of an established not-for-profit organisation (civil society or other non-governmental organisation) and may therefore be subject to additional site-specific hiring criteria. For the purposes of SUCCEED, all providers and supervisors must have the literacy, numeracy and fluency in the local language required to successfully complete training and carry out their responsibilities related to the intervention. Candidates who have completed the SUCCEED training and go on to deliver the intervention are eligible for inclusion in a competency assessment and inclusion in interviews, regardless of whether they were able to continue delivering the intervention for the duration of the pilot. (This is to allow for discussion of barriers which may prevent providers or supervisors from carrying out their roles.) Further eligibility criteria specific to each participant type are discussed further below.

#### People with lived experience of psychosis

For people with lived experience of psychosis, we will include adults who have a current or past diagnosis of schizophrenia or other primary psychotic disorder, bipolar or depressive disorder with psychotic symptoms, or a maternal mental health or behavioural disorder with psychotic symptoms, as per the World Health Organisation’s International Classification of Diseases (Version 11) [39]:

- Schizophrenia (6A20)
- Schizoaffective disorder (6A21)
- Schizotypal disorder (6A22)
- Acute and transient psychotic disorder (6A23)
- Delusional disorder (6A24)
- Other specified (6A2Y) or unspecified (6A2Z) primary psychotic disorder
- Bipolar type I disorder with psychotic symptoms (6A60.1, 6A60.5, 6A60.7, 6A60.A)
- Bipolar type II disorder with psychotic symptoms (6A61.3, 6A61.5)
- Single episode depressive disorder with psychotic symptoms (6A70.2, 6A70.4)
- Recurrent depressive disorder with psychotic symptoms (6A71.2, 6A71.4)
- Mental or behavioural disorder associated with pregnancy, childbirth or the puerperium, with psychotic symptoms (6E21)

We will exclude secondary psychotic syndromes (6E61), as these are considered to be the direct consequences of physical health conditions as opposed to mental health conditions. We will also exclude those diagnosed with a substance-induced psychotic disorder (6C40.6-6C47.6), as recovery from substance use conditions is a specialist area outside the scope of the SUCCEED intervention under development. Finally, we will exclude people who are currently homeless, for logistical reasons; the SUCCEED intervention relies on PSWs and CSWs being able to regularly contact participants, including for home visits. People affected by psychosis who do not have an eligible family member/carer are not excluded.

#### Family members

We will invite a family member of each participant with lived experience (see above). The family member should be a relative (e.g. adult child, sibling, cousin, spouse, parent, aunt/uncle, grandparent), ideally sharing a house/homestead with the participant and identified as being closely involved in their care (e.g. supporting activities of daily living, treatment). We exclude family members whose support is exclusively financial, as SUCCEED will most benefit those with direct involvement in day-to-day activities.

#### Community support workers

In addition to the general criteria described above, for the purposes of SUCCEED, CSWs must have a minimum of 3 years of secondary education, live locally and have established relationships and sufficient knowledge of the local community to understand the different resources available.

#### Peer support workers

For the purposes of SUCCEED, PSWs must have lived experience of psychosis and be “on the road to recovery”, such that they are able to cope with the basic demands of the work (though it is understood that further support may well be required at times, and reasonable accommodation will be given where necessary). Practically, PSWs must live in close enough proximity to the study area to carry out their duties effectively. We expect PSWs will have at least a secondary school level of education or equivalent, but candidates will not be excluded on the basis of educational attainment.

#### Supervisors

Supervisors are people in managerial positions within the organisation hosting the SUCCEED intervention who are accountable for the delivery of the intervention. For the purposes of SUCCEED, supervisors must have completed secondary education.

#### Data collectors

SUCCEED data collectors involved in the baseline assessment of WHOQOL-BREF will be eligible for interviews about their experiences administering the tool. SUCCEED data collectors are local university staff subject to eligibility requirements specific to their academic grade. For the purposes of SUCCEED, all data collectors must have a university-level or postgraduate education; be sufficiently literate, numerate and fluent in the local language to assist participants in completing the WHOQOL-BREF assessment; have completed SUCCEED training in research ethics and in the administration of the WHOQOL-BREF instrument.

### Intervention

The three key components of the SUCCEED intervention are peer support, case management and livelihoods activities, which are delivered by a gender-mixed team including one PSW and one CSW, through a combination of individual visits and self-help group meetings. As described above, the PSW is a person with lived experience of psychosis who leverages their experiences of illness and recovery to support others. Although peer support is a flexible intervention, SUCCEED PSWs are trained on a variety of manualised tools and techniques adapted from previous studies (e.g. Brain Gain II project, UPSIDES trial) [30, 40] and normative guidance (QualityRights) [41] which they may choose to employ where relevant (Table 3). The CSW focuses on mobilising families and communities to activate resources in support of participants to access their rights (e.g. education and employment opportunities, social and recreational activities), drawing on established models of mental health case management and community-based inclusive development (e.g. RISE trial, CBM Global’s community mental health model, WHO CBR Guidelines) [21, 25, 26, 42–44].

**Table 3:** Description of tools and techniques included in SUCCEED peer support training

Circles of safety	Technique to aide PSWs and peers in identifying their own boundaries and deciding what of their lived experience they are comfortable sharing, with whom and in which contexts
Tree of life	Reframing a peer’s personal narrative, emphasising growth and resilience through personal challenges
Recovery planning	Using structured tools to consider life goals, personal wellness, and advance planning for situations when a peer is becoming unwell
Accompaniment	Accompanying peers and family members to appointments, for example with healthcare providers or school administrators, to help advocate for peers’ needs
Shared decision-making	Facilitating participatory discussions with clinicians on treatment options (especially in terms of side effects and management) when accompanying peers to clinical appointments (see above)

Individual visits may take place in participants’ homes, clinical settings, other community settings or remotely via telephone. PSWs and CSWs run face-to-face self-help groups for people with lived experience and their family members, respectively, at an agreed meeting place. As in other models of mental health and development (e.g. BasicNeeds) [45], self-help group meetings create a platform for livelihood activities, which in this case will use an asset-based community development (ABCD) [31] approach focused on harnessing communities’ existing strengths and potential. SUCCEED PSWs and CSWs may also encourage informal peer support, for example by setting up WhatsApp groups for members of self-help groups to interact outside of meetings, encouraging peers from the same religious denomination to attend church or mosque together, requesting peers to accompany each other on errands, or by arranging recreational activities like sports days or gardening.

Both the PSW and CSW are managed by a supervisor embedded within an existing partner organisation (a non-governmental or civil society organisation with a track record of disability-inclusive health and development work in-country). All providers and supervisors complete a standardised 2-week training combining a series of core modules with role-specific modules. Further details of the intervention and its implementation are provided below, in Table 4, and in the SUCCEED Intervention Manual and training materials (available upon request).

**Table 4:** Key features of the Implementation of the SUCCEED Intervention

Staff recruitment
• PSW, CSW and Supervisor may be existing staff within the partner organisation or recruited as new staff for the duration of the SUCCEED intervention • Recruitment can be through open advertisement, or local organisations • Recommendations may be particularly helpful for the identification of potential PSW candidates • The SUCCEED Intervention Manual outlines essential and desirable criteria for each role • Consideration of the gender-mix of PSW-CSW teams, languages spoken, etc. is important • In addition to formal training, PSWs and CSWs receive additional orientation to SUCCEED and to the partner organisation as part of the onboarding process
**Capacity-building**
The SUCCEED training was developed collaboratively by SUCCEED research teams from all four countries as well as the UK coordinating centre. Teams consist of multidisciplinary academic, clinical and peer researchers. The training comprises 10 core modules combined with nine role-specific modules directed at PSWs, CSWs and supervisors, respectively Core modules include: 1. Introduction to training, SUCCEED intervention, recovery and principles of CBR 2. Introduction to psychosis/mental health and lived experience 3. Rights-based approaches and shared decision-making 4. Safeguarding, managing risk and unexpected circumstances 5. Essential care and practice 6. Counselling skills, building rapport and facilitating groups 7. Livelihoods activities 8. Leadership, collaboration and managing conflict 9. Monitoring and evaluation 10. Bringing it all together: review and reflection • Facilitators employ a variety of methods such as didactic teaching, live demonstration and role-play, individual and group work, and both small- and large-group discussion • Although some multi-media resources are included (e.g. video links), the training is intended to be delivered in a face-to-face format over a 2-week period by members of the SUCCEED research team, with at least one facilitator having lived experience of psychosis (typically, a peer researcher) • The training schedule accounts for breaks between role-specific modules for paced learning and to protect time for self-study • As described further below, an adapted version of the ENhancing Assessment of Common Therapeutic factors (ENACT) tool [46] is used to assess providers’ competencies at endline • All training materials are translated and adapted to the local context where appropriate, and are available upon request to the study authors
**Supervision**
• A trained supervisor embedded within the partner organisation is responsible for supervising the CSW and PSW and serving as the main liaison between the research team and the partner organisation for issues related to intervention delivery • The supervisor is expected to maintain close regular contact with the CSW and PSW (e.g. daily phone calls), observe one intervention activity each week and arrange biweekly meetings (preferably face-to-face) • Supervision sessions should cover job performance and trouble-shooting as well as more general discussion of wellness and wellness planning, which may be especially important for emotionally demanding roles • Supervisors are responsible for ensuring that all staff operate in line with the SUCCEED code of conduct and that any safeguarding concerns, potential adverse events and other challenges are reported promptly and following SUCCEED protocols (see “Monitoring” and “Risk management”)
**Monitoring and evaluation**
• SUCCEED’s monitoring and evaluation (M&E) and reporting procedures serve a dual purpose, generating important research data while also promoting quality assurance over the course of the pilot • PSWs and CSWs complete a M&E form at each visit and self-help group meeting • Supervisors attend one intervention activity each week, completing a semi-structured observation form that can then be discussed with providers in supervision sessions • Once a month, a member of the SUCCEED research team attends the same activity and completes their own form, to check the validity of the supervisor’s observations • Supervisors also prepare a monthly reporting form compiling information from the CSWs’ and PSWs’ M&E forms and the semi-structured observation form, and indicating whether there are any concerns that should be discussed with the research team
**Compensation**
• PSWs, CSWs and supervisors are expected to have formal contracts with appropriate compensation in line with the human resources policies and procedures of the host organisation • Compensation should reflect the qualifications of the CSW and the PSW while recognising the added value of the PSW’s lived experience (which may not be reflected in educational attainment or other certifications typically used to determine salary level) • Any further materials required for delivery will be provided by the host organisation or research team

#### Adherence

As this is a pilot study, fidelity of intervention delivery and adherence by participants with lived experience of psychosis and family members are important indicators of feasibility and acceptability that we seek to capture through routine M&E, semi-structured observations and reporting (described further below in “Routine monitoring and evaluation” and in Table 5)—which also offer opportunities for supervisors to identify and resolve any quality assurance issues on an ongoing basis. Participants with lived experience of psychosis are expected to receive one initial home visit (or alternative location if preferred) within 2 weeks of recruitment, followed by (at minimum):

1. Four monthly face-to-face visits with PSW plus four monthly follow-up contacts (which may take place face-to-face or remotely)
2. Four monthly face-to-face visits with CSW plus four monthly follow-up contacts (which may take place face-to-face or remotely)
3. Four monthly PSW-facilitated self-help group meetings with livelihood activities

**Table 5:** Assessment of WHOQOL-BREF embedded in pilot study

**Topic**	Timepoint		Participants			**Methods**
	**Baseline**	**Endline**	**Participants with lived experience of psychosis**	**Family members**	**Data collectors**	
Face validity	X		X	X		Cognitive interview immediately following completion of WHOQOL-BREF
Validity of completion by proxy	X		X	X		Comparison of WHOQOL-BREF completed by participant with lived experience versus family member
Acceptability and feasibility	X				X	Interview following completion of baseline for all participants
		X	X	X		Questions related to WHOQOL-BREF integrated into endline focus group discussions
		X			X	Analysis of research process data recorded by data collector (time taken to administer, proportion of participants who complete all WHOQOL-BREF questions)

Family members enrolled in the pilot will also be offered four monthly self-help group meetings with livelihood activities facilitated by the CSW.

### Outcomes

#### Primary outcome

The primary outcome for the before-and-after study is improvement in the quality of life of adults with lived experience of psychosis, assessed via WHOQOL-BREF (abbreviated World Health Organisation Quality of Life questionnaire) administered at baseline and endline. WHOQOL-BREF is a 26-item measure evaluating the individual’s perception of their health and wellbeing over the previous 2 weeks [47]. It is designed to be self-administered, though it is increasingly being administered by proxy (e.g. caregiver assessment) for situations in which participants are too unwell or the tool proves too burdensome for self-completion [48]. It covers four domains (physical, psychological, social relationships and environment), and each item is answered on a 5-point Likert scale, where 1 indicates “not at all” and 5 indicates “a large amount”. The total score across these items is then transformed onto a scale of 0–100, with higher scores indicating better quality of life [49]. While WHOQOL-BREF is widely used internationally, with over 100 culturally adapted translations to-date [50], it may not adequately capture the cultural nuances and specific experiences of participants across all four SUCCEED countries. Hence, additional methods will be used to evaluate WHOQOL-BREF’s performance as a measurement tool for the pilot in preparation for larger-scale research, as described below in Table 5.

#### Other outcomes

We use Proctor et al.’s [35] framework to structure our investigation of other client-level, service-level and implementation outcomes. These are mainly assessed qualitatively or through a combination of qualitative methods and analysis of routine M&E data, as described further below.

##### Client-level outcomes

Besides quality of life, the primary outcome describes above, the main client-level outcome under investigation is satisfaction (which can also serve as an indicator of acceptability). Questions regarding the satisfaction of participants with lived experience of psychosis and their family members are integrated into focus group discussion guides, with the aim of identifying common themes to be integrated into a structured satisfaction survey for a more systematic assessment of satisfaction as part of SUCCEED’s future research.

Service outcomes included in Proctor et al.’s (2011) framework are adapted from the Institute of Medicine’s Standards of Care and include the following: efficiency, safety, effectiveness, equity, patient-centredness and timeliness [35]. For the pilot, questions regarding all seven of these outcomes are integrated into discussion guides for focus groups and interviews. We will also use quantitative methods to investigate the following:

- Safety: The number and type of serious adverse events reported over the course of the study.
- Effectiveness: Change in WHOQOL-BREF (see above) for participants with psychosis, between baseline assessment and four-month follow-up.
- Equity: Number of participants with lived experience of psychosis (and family members) recruited and percentage who drop-out, disaggregated by gender, marital status, number of children, religious affiliation, ethnicity, level of education and employment status.
- Timeliness: Average time between recruitment and initiation of services, and average time between contacts with service providers, according to M&E data.

##### Implementation outcomes

We will assess early- to mid-stage implementation outcomes (acceptability, appropriateness, feasibility, fidelity) mainly through qualitative research. As above, this will include focus groups and interviews, as well as semi-structured observation of intervention activities by supervisors and research staff. For further assessment of fidelity, we will also use M&E data to investigate whether thresholds for minimum number of visits and group meetings are met (see “Adherence”) and assess competencies of providers at endline (see Table 4, “Capacity-Building”) using the ENACT tool.

#### Provider competency

The competencies of SUCCEED PSWs and CSWs will be assessed using an adapted version of the ENhancing Assessment of Common Therapeutic factors (ENACT) tool [51] administered at the study’s endline. ENACT measures a set of skills (referred to as common factors) required for task-shared mental health care delivered by non-specialists (e.g. non-verbal and verbal communication, collaborative processes, rapport). For the purposes of SUCCEED, the adapted ENACT tool will be applied through standardised role plays covering a range of common presentations of psychosis, which were developed by a multidisciplinary team of SUCCEED researchers and adapted to their local contexts. The scenarios acted out through role-play attempt to capture both clinical and other aspects of the lived experience of psychosis for those directly affected as well as their family members. Common factors are organised into 18 domains and rated on a three-point Likert scale (1 = “needs improvement”, 2 = “done partially”, 3 = “done well”) [52]. Ratings are calculated as a sum across all domains with higher scores indicating a higher level of competency across the common factors.

### Participant timeline

**Fig. 1:**
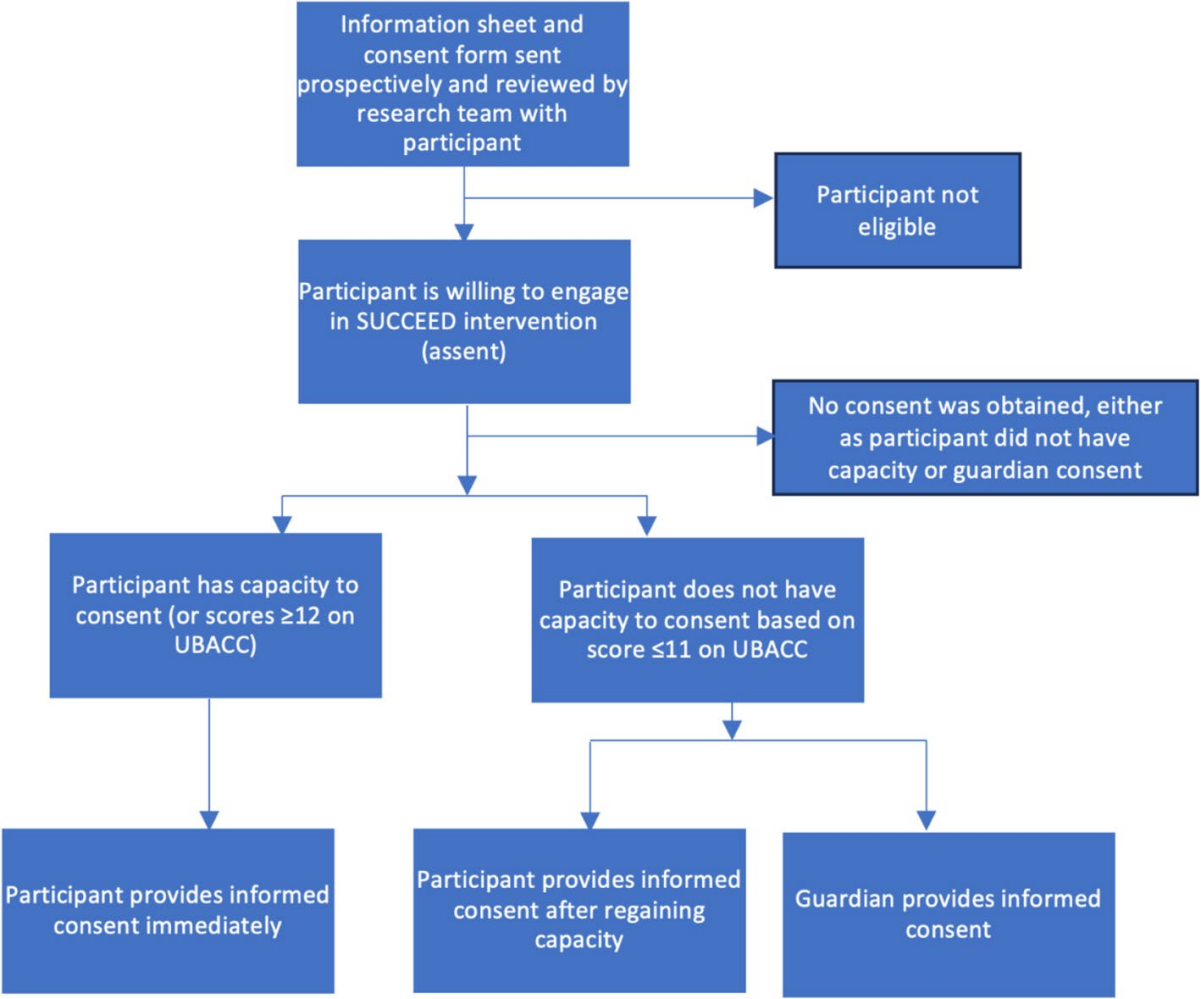
Flow chart for securing informed consent where capacity is unclear

The intervention will run for 4 months of a 6-month study period. The providers and supervisors will be recruited and trained during an initial month-long preparatory phase. During the recruitment phase, which will also last approximately 1 month, potential participants with lived experience of psychosis and family members will be identified as described above. Consent will be secured at this time, and the WHOQOL-BREF and cognitive interview will be administered. The data collector responsible for administering the WHOQOL-BREF will participate in an interview at the end of the recruitment/baseline stage. The CSW and PSW will then undertake their first visits to participants’ homes, during which participants with lived experience of psychosis and their family members will be invited to their first self-help group meetings. Delivery and monitoring continue through months 3–5 (see “Intervention”). In month 6 (endline), final face-to-face visits are conducted by the PSWs and CSWs to close the intervention. Endline quantitative (WHOQOL-BREF) and qualitative data (focus groups, interviews) are also collected, and providers complete the ENACT assessment (Table 6). See Fig. 1 for the SPIRIT schedule of enrolment, interventions and assessments.

**Table 6:**
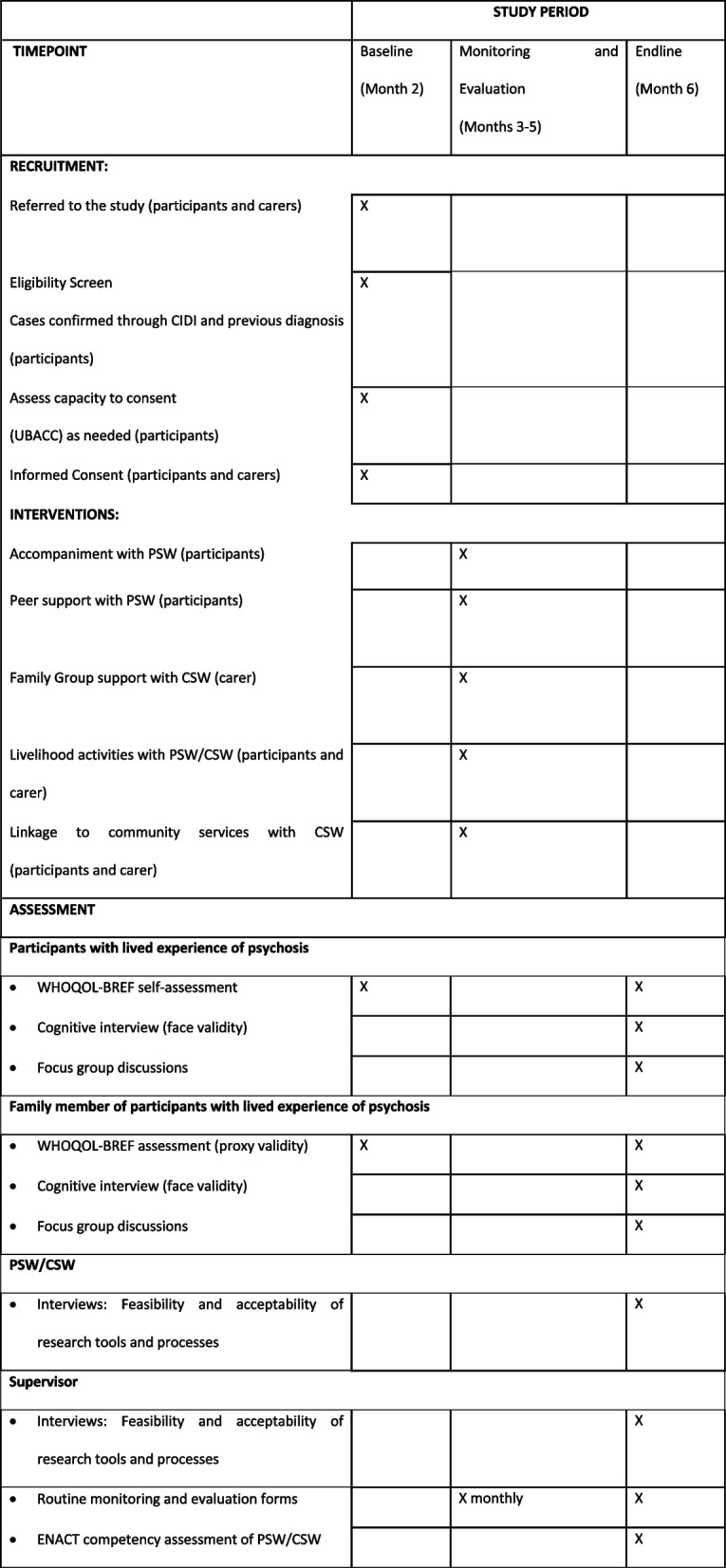
Schedule of enrolment, interventions and assessments

### Sample size

For each of the four SUCCEED sites, we expect to recruit 10 people with lived experience of psychosis and up to 10 family members (one for each participant with lived experience of psychosis, although participation of a family member is not mandatory). We will also include the PSW, CSW and supervisor involved in delivery, as well as the data collector(s) involved in administering the WHOQOL-BREF at baseline. Across the four sites, this would yield approximately 96 participants, in total.

For the purposes of the pilot, we do not expect to recruit a representative sample of people with psychosis; rather, we will purposively select a sample of 10 people with psychosis, aiming to maximise variation across a range of sociodemographic (described below) and clinical (diagnosis) characteristics to better ensure a diverse representation. This is to aid in identifying any unforeseen barriers that may affect willingness or ability to participate in the intervention.

In determining the sample size, the need for variation is weighed against several practical considerations: (1) what the SUCCEED research team felt would be a reasonable caseload for the frontline providers involved in delivery; (2) a desirable group size for livelihoods and self-help activities; (3) a desirable group size for subsequent focus group discussions, for research purposes. We also note that this sample size is in line with those of similar studies piloting CBR for schizophrenia (“RISE” as described in Asher, et al. 2018) [26] and peer support for people with severe mental health conditions, including psychosis (“UPSIDES” as described in Puschner et al. 2019) [40] in sub-Saharan Africa. Both studies recruited 10 participants with mental health conditions; RISE recruited “dyads” with one family member for each participant with a mental health condition.

### Recruitment

Potential participants with lived experience of psychosis and their family members will be recruited through referral from health services and community identification activities (described below). Where a diagnosis cannot be confirmed from health records, research workers will be trained to assess whether potential participants meet the criteria for psychosis using the World Health Organisation’s Comprehensive International Diagnostic Interview (CIDI) screening tool available in English and translated into the main local language at each study site [53].

### Health services

Primary, secondary and/or tertiary health care facilities may serve as sources of recruitment, depending on the services available in the study site under consideration. Either the CSW or a research worker will approach staff to refer patients who may have psychosis to the study. Staff will be given an informational letter for their use, as well as copies of invitation letters and information sheets for potential participants. Invitation letters will include contact details for team members responsible for recruitment, to arrange an appointment to discuss the information sheet and consent form.

#### Community settings

In community settings, the SUCCEED CSW and PSW should both be knowledgeable of local communities and able to help identify and engage with people with psychosis in the area. Additionally, the CSW will arrange a Community Mental Health Forum (CMHF), which doubles as an awareness-raising activity as well as an opportunity to advertise the study and describe the types of participants that SUCCEED is looking to recruit. The CSW will present an adapted version of the Community Informant Detection Tool (CIDT), which provides a brief narrative and pictorial description of a typical case of psychosis, for use in community settings. Members of the CMHF can then follow-up directly with the CSW for invitation letters with the study team’s contact details to arrange an appointment to discuss the information sheet and consent form.

#### Other participants

For the endline, providers (PSWs, CSWs) and supervisors will be approached by a research worker external to their organisation, for consent to participate in an ENACT assessment and an interview. Data collectors will be approached by another SUCCEED research worker, independent of their line management, for consent and an interview. Providers, supervisors and data collectors will be reassured that they are under no obligation to participate, that they have the permission of their employers to participate during working hours and that their responses will in no way affect their employment.

##### Data collection

**Baseline**

The paper-based enrolment form used for people with lived experience of psychosis and family members collects essential sociodemographic data (age, gender, marital status, number of children, level of education, employment, religious affiliation, ethnicity), clinical information (prior diagnosis, if applicable) and detailed information on how to contact participants, including their preferences regarding face-to-face contact, and should take 20 min or less to complete. As described above, participants with lived experience of psychosis who are enrolled in the study then complete a paper-based WHOQOL-BREF (see “Primary outcome”). If the participant also has an eligible family member enrolled in the study, the family member will be asked to complete a proxy assessment. WHOQOL-BREF may be self-administered or administered in an interview format, and is estimated to take 30 min or less to complete. Upon completion, the participant and family member complete cognitive interviews following a semi-structured interview guide developed for the purposes of SUCCEED. The enrolment and WHOQOL-related data collection are carried out by a trained data collector who, upon completion of baseline data collection, will be asked to participate in an interview by another researcher following a semi-structured interview guide also developed for SUCCEED to assess WHOQOL-BREF as a study measurement tool. Interviews take approximately 1 h, with both an interviewer and a note-taker present. Interviews are audio-recorded and transcribed, with permission.

#### Routine monitoring and evaluation

PSWs and CSWs complete a monitoring and evaluation (M&E) form at each contact with participants (home visit, self-help group meeting, etc.). M&E forms are designed to be short (15 min to complete, or less) and collect basic process data on the service provided (e.g. location and duration of contact, who participated, what tools/topics were covered).

The supervisor will also carry out semi-structured observation of intervention delivery. Supervisors will be requested to observe at least one intervention activity each week, using a semi-structured observation form to record insights into the providers’ performance, fidelity to the intervention manual, and the engagement of the participants (which should take no more than 30 min). For quality assurance, a SUCCEED researcher will also attend one intervention activity each month and record their observations using the same guide. Over the course of the 4-month follow-up period of the pilot, SUCCEED researchers should plan to record observations on at least one of each of the following:

- Facility visit (recruitment and/or accompaniment of a participant to health services)
- Community Mental Health Forum
- Self-help group meeting
- Livelihoods activity

Data from M&E forms and semi-structured observations will be aggregated along with research administrative data (e.g. participant drop-outs) in monthly reports prepared by the supervisor at the host organisation for the SUCCEED research team. The SUCCEED research team may carry out quality checks, if indicated, to ensure that monthly reports are complete and there are no obvious errors.

#### Endline

A WHOQOL-BREF self-assessment for participants with lived experience of psychosis is repeated at approximately 4 months after recruitment, following the same procedures described above (see “Baseline”).

An endline ENACT competency assessment is also completed for SUCCEED providers (PSWs, CSWs) using an adapted version of the ENACT tool applied by a trained SUCCEED research worker and supervisor to assess trainee competencies (see “Provider competency”, above). The assessors will record their ENACT scoring for each provider on a paper-based form during a standardised role-play (estimated to last 15–20 min) [54]. The mean score between the two assessors will be calculated for each item and summed to provide an overall score (though we do not assign any a priori cut-off to determine competence level).

Endline qualitative data collection comprises interview and focus group discussions following semi-structured discussion guides tailored to each participant type. Interviews will take approximately 1 h, and each focus group will take approximately 2 h to complete. Both interviews and focus group discussions will have a trained facilitator and note-taker present and will be audio-recorded and transcribed, with permission. Facilitators will also be instructed to take notes immediately after each interview or focus group to jot down key points that surprised them, were in contradiction to the way in which they think about the topic at hand or affirmed a particular viewpoint, to contribute to the subsequent data analysis.

### Data management

Hard copies of data collection tools and notes will be kept in locked cabinets, and soft copies (i.e. audio recordings and transcripts) will be kept on secured, password-protected laptops that can only be accessed by SUCCEED researchers. All research equipment, hard and soft copies of data will be kept in locked offices. At least one researcher per SUCCEED study site has been nominated as a data manager and undergone additional training in data management provided by an experienced statistician [55]. All recordings will be destroyed once transcription has been completed and checked. With the exception of shared data deposited in repository,^2^ all research data will be destroyed 10 years after the completion of SUCCEED’s programme of research (April 2036), in line with LSHTM guidelines, unless local regulations stipulate otherwise.

Quantitative data will be collected using predesigned paper-based data collection tools and anonymised before entry. Data will be double-entered into a customised electronic data collection form with pre-programmed data checks. Further checks of range, consistency and completeness will be carried out, and data queries will be raised to clean the data. The data will be hosted on an LSHTM server and protected using asymmetric encryption keys. A codebook will list all variables and value labels along with a description and details on the tool and question from which they were derived.

Key informant interviews and focus group discussions will be captured via audio recordings, with participants’ consent. (An interviewee may request that the interviewer take handwritten notes, as opposed to a recording, though this is not feasible for focus group discussions.) All recordings and notes will be confidential and shared only among research team members. All interviews and focus group discussions will be anonymised immediately upon transcription (discussed further below). All recordings will be destroyed once transcription has been completed and checked. A separate list of participants will be held securely at each site, to allow the return of findings to participants.

### Data analysis

Quantitative data will be analysed using Stata statistical software [56]. Descriptive characteristics of study participants will be reported. WHOQOL-BREF total scores will be calculated, and we will report endline score adjusting for baseline score to determine improvement over time. The scores of self-completed and proxy-completed questionnaires will be compared graphically to assess their correlation, and the validity of proxy-completed WHOQOL-BREF will be investigated against self-completion as the gold standard. Qualitative interview and focus group transcripts will be analysed in ATLAS.ti [57] using thematic analysis, following a process of coding of themes and grouping into categories. A framework analysis approach [58, 59] will be adopted to develop a standardised coding frame that can be used across all countries to allow cross-country comparison of the results. The coding frame will be sufficiently flexible to allow addition of emerging country-specific themes during the analysis. Each SUCCEED country team will be responsible for coding their own data, led by the Country PI. Each focus group discussion or interview will be analysed by two coders. Regular meetings will bring together country teams with LSHTM team to discuss progress of coding.

All qualitative data will initially be analysed in the local language to avoid losing information and nuances specific to local language in the translation processes. However, the multi-site, cross-country nature of this study will necessitate translation of all transcripts into English. Where analysis in a local language is not possible, or for cross-country analyses of the data, analyses will be carried out in English with input from the country team members to check appropriateness of analysis and interpretation.

### Ethics and dissemination

#### Ethical approvals

Institutional approval was received from the London School of Hygiene and Tropical Medicine Research Ethics Committee in the UK, the coordinating centre of the SUCCEED consortium (Ref 28,346). Local ethical approval was received in Malawi through the Kamuzu University of Health Sciences, College of Medicine Research and Ethics Committee (COMREC) (Ref Sefasi P/O3/23/4032), in Nigeria through the University of Ibadan/University College Hospital Ethics Committee and Oyo State Research Ethical Review Committee (Ref NHREC/05/01/2008a), Ministry of Health, Ibadan, Nigeria; in Sierra Leone through the Office of the Sierra Leone Ethics and Scientific Review Committee, Directorate of Training and Research, Ministry of Health and Sanitation (Ref SLESRC No:018/03/2023); and finally in Zimbabwe through the Medical Research Council of Zimbabwe and Research Council of Zimbabwe (Ref MRCZ/A3015). Additional details on the ethical considerations and procedures for this study are available from the authors upon request.

#### Informed consent

Experienced researchers from SUCCEED’s in-country research teams will be responsible for taking consent. All SUCCEED researchers are trained in research ethics. Wherever possible, the information sheet and consent form will be provided to prospective participants at referral or during their initial contact with the research team, leaving at least 24 h to consider their decision. Consent will be secured before data collection can commence. The researcher will remind potential participants about the research and share hard copies of the informational letter in English or a local language, as needed. The potential participants will be invited to ask any questions before providing consent. If agreed, they will sign the consent form. For interviews and focus group discussions, the information sheet will clearly specify the expectation and/or requirement to audio-record the session. The consent form will also ask for permission to audio-record the discussion. (An interview may take place without audio-recording; however, for focus groups, this will not be possible.) Participants invited to a focus group who do not wish to be audio-recorded will be given the option of participating in a private interview instead. Additional procedures are described below for low literacy and for situations in which capacity to consent is unclear.

#### Low literacy

All potential participants are asked at the beginning of the consent process whether they are comfortable reading and writing in either English or a local language. For those with low literacy, an impartial witness will be identified to assist the potential participant in the consent process. A standard recording of the translated participant information sheet will be available in the most commonly spoken local language at each study site. This will be played aloud for the potential participant in the presence of the impartial witness and the researcher, who will be available to answer any questions. The potential participant will sign with a thumbprint or a symbol (X). The impartial witness must then sign to attest that the study information has been presented to the participant as per the information sheet. The researcher must also sign to attest that they have explained the study information accurately and that it was understood in the presence of the impartial witness.

#### Capacity to consent

All participants must assent before any data can be collected. We take the position, in alignment with the UK Mental Capacity Act (2005) [60], that people are presumed to have capacity to consent unless there is evidence to the contrary. Should there be any reason to believe that a potential participant is unable to provide free and informed consent (related to ability to comprehend, retain, communicate or voluntarily act on, information), then the researcher will apply an adapted version of the University of California San Diego’s Brief Assessment of Capacity to Consent (UBACC) [61]. To apply UBACC, the researcher reviews the information sheet and consent form with the potential participant, then asks a series of questions about their content and implications, scoring each response on a Likert scale ranging from 0 to 2. A UBACC score of 11 or below suggests that a potential participant does not have capacity to consent. However, if a potential participant scores a 12 or higher, the researcher must still use their best judgment and seek a second opinion if needed to confirm eligibility based on capacity to consent. If after completing the UBACC it remains unclear whether a potential participant has capacity (or if the potential participant clearly does not have capacity), guardian consent can substitute. Alternatively, if an assenting participant regains capacity over the 4-month follow-up period, they can provide (or withdraw) consent at any time, overriding their guardian’s consent. See Fig. 1 for details.

#### Risk management

Participants will not be subject to any medical procedures as part of this research. All participants, apart from those with lived experience of psychosis and their family members, will be asked about their perspectives and practices rather than any personal information about themselves. However, participants with lived experience and their family members may be asked personal questions related to mental health and recovery, which can be uncomfortable at times to discuss. Participants are reassured in all informational materials that they may skip questions or withdraw from the study at any time, without any negative consequences. Data collectors will be trained in looking for signs of distress and, when necessary, will pause the session to ask if they are okay and remind them that they can stop or withdraw from the study. When necessary, the session will be stopped and information on who they can talk to for assistance/support will be provided.

Each SUCCEED study site is staffed by a multidisciplinary team including mental health professionals. A clear procedure will be put in place where any concern identified by the SUCCEED researcher engaging with any participant will be communicated with the Programme Manager in the country site, who will then be responsible for arranging follow‐up by a mental health professional. Each SUCCEED research team includes one or more mental health professionals qualified to provide psychosocial counselling and low‐ intensity psychotherapies, and to refer complex or unresponsive cases to specialist care, if desired. Each study site has already made a formal agreement with at least one specialist mental health service willing to accept referrals from the study. However, as SUCCEED actively promotes shared decision-making over coercive care, the participant’s (and family members’, if appropriate) preferred sources of support will ultimately determine the referral process.

For monitoring purposes, a Trial Management Group has been established with the following membership: The UK-based SUCCEED CEO; the International (UK-based), West African (Nigeria) and Southeast African (Zimbabwe) Research Directors; the UK-based SUCCEED Research Manager and Trial Manager; the UK-based statistical advisor and Zimbabwe-based statistician; and all other Local Principal Investigators (Malawi, Sierra Leone) and Project Managers from each SUCCEED country. The Trial Management Group will meet on a monthly basis to review the progress of the pilot study, including a summary of the information collected via supervisors’ monthly reporting forms (see “Monitoring and evaluation”, above). If a potential adverse event or safeguarding issue is raised, the CEO will convene the Trial Management Group within 48 h to investigate and determine the appropriate course of action, including further reporting to local and international ethics committees, in line with SUCCEED’s Safeguarding Policy, guidance provided by LSHTM’s office of Research Governance and Integrity, and local regulations. As there are only two time-points for the collection of outcome data, no interim data analysis will be performed, and no audits are planned.

#### Compensation

Participants will receive modest stipends for participation in data collection activities (i.e. baseline/endline assessments, focus groups, interview discussions). Stipends ($10 USD equivalent per data collection event) are based on SUCCEED’s policy for subsistence rates in each country, aligned with the UK Foreign Commonwealth and Development Office’s guidance, and seek to reasonably compensate people for their time without unduly influencing their decision to participate. This stipend reflects lost earning as a result of participation in the study and/or additional costs, such as travel. Appropriate refreshments, where relevant, will also be provided (e.g. for focus groups).

#### Dissemination

All academic publications will be made available in Open Access, either by the journal or through LSHTM’s online repository [62]. SUCCEED publications are accompanied by lay summaries and targeted communications plans developed and executed by the consortium’s cross-site Communications team. These will include plans to communicate findings with the host organisation delivering the intervention and with study participants.

## Discussion

This pilot study offers an important opportunity to assess and improve upon the SUCCEED Africa intervention and research methods, tools and processes, before these are scaled up to power two randomised controlled trials (Nigeria, Zimbabwe) and further process evaluations (Malawi, Sierra Leone). The pilot study aims to test the feasibility, acceptability and preliminary outcomes of the SUCCEED community-based intervention for people with lived experience of psychosis in four sub-Saharan African countries. Employing an uncontrolled before-and-after study design, the pilot assesses changes in quality of life and other key outcomes over a 4-month period. This pilot will provide critical insights and practical knowledge to inform the larger-scale evaluation of the intervention.

The consortium’s commitment to co-production, including the involvement of peer researchers with lived experience embedded within local research teams and the oversight of a cross-consortium Lived Experience Advisory Panel (LEAP), is a strength of the formative research and other activities informing the design of this pilot (e.g. Theory of Change workshops, literature reviews, situation analyses, local stakeholder meetings). The intervention itself incorporates a formal peer support component, again reflecting a commitment to lived experience involvement. Further, delivery of the intervention by local organisations of persons with disabilities (OPDs) is expected to promote sustainability, while also building OPDs’ capacity in the area of mental health and psychosocial disability—an under-represented issue on most disability rights agendas [63, 64].

However, critics have argued that co-production and other seemingly progressive approaches to promoting involvement in health research are not risk-free [65]. Indeed, reflections recently published by a lived experience working group involving several SUCCEED Africa members highlight the broader geopolitical context of global mental health, differing levels of research experience, different stakes in and resources for conducting research, among other challenges in carrying out an effective international research collaboration on psychosis [66]. In the context of the SUCCEED Africa pilot, representation may be an especially important limitation. Peer researchers and other LEAP members tend to be educated professionals living in urban areas with access to specialist mental health care, while our research focuses mainly on underserved communities. Additionally, contextual differences among the participating countries may affect the acceptability and feasibility of the intervention. For example, variations in terms of the stigmatisation of and beliefs about mental health, and community support structures, could influence participants’ engagement with the intervention. The in-depth qualitative research and consultation that preceded the pilot were designed to help bridge these gaps; however, we expect the pilot to uncover many unforeseen issues that will need to be addressed before progressing to larger-scale implementation and evaluation. Findings will be relevant not only to SUCCEED, but also to others interested in promoting a rights-based approach to community mental health for people with psychosocial disabilities in low-resource settings. 

### Supplementary Information

Additional file 1: Cross-Site Theory of Change

Additional file 2: SPIRIT Checklist

## Data Availability

Study materials are available upon request.

## Abbreviations

YLDs: Years lived with disability
DALYs: Disability-adjusted life years
LMIC: Low- and middle-income countries
WHO: World Health Organisation
CBR: Community-based rehabilitation
RPC: Research Programme Consortium
ToC: Theory of Change
ABCD: Asset-Based Community Development
WHOQOL-BREF: World Health Organisation Quality of Life Brief Version
M&E: Monitoring and evaluation
SPIRIT: Standard Protocol Items: Recommendations for Interventional Trials
TIDieR: Template for Intervention Description and Replication
CSW: Community support workers
PSW: Peer support workers
CIDI: Comprehensive International Diagnostic Interview
CMHF: Community Mental Health Forum
UBACC: University of California San Diego’s Brief Assessment of Capacity to Consent
ENACT: The ENhancing Assessment of Common Therapeutic factors
CIDT: Community Informant Detection Tool
CBM: Christian Blind Mission
LEAP: Lived Experience Advisory Panel
OPD: Organization of Persons with Disabilities

## Footnotes

1 As we discuss elsewhere, psychotic symptoms may also occur in individuals with other mental and neurological disorders or in the absence of any diagnosable disorder. The term “psychoses” encompasses experiences of psychosis regardless of the presence or absence of a diagnosable psychotic disorder. For accuracy, we use “psychotic disorders” as necessary to discuss relevant literature or methods specific to this diagnostic category. However, as a consortium, we have registered our discomfort with deficit-based language in mental health research and are working to develop consensus-based guidelines for future publications.

2 SUCCEED’s Data Access and Publication Policy stipulates that data are jointly owned by all SUCCEED partnered institutions: University of Ibadan, University of Makeni, Kamuzu University of Health Sciences, and the University of Zimbabwe; thus, all partners are free to access anonymised SUCCEED data. Access is facilitated by the Data Management Team, which is comprised of nominated researchers at each SUCCEED site, who receive additional training and support on data management as part of the SUCCEED Capacity-Building workstream. The policy also describes the process by which any individual internal or external to SUCCEED may submit an expression of interest using a standardised online form outlining their intention to access and/or publish SUCCEED data; expressions of interest are reviewed on a regular basis by SUCCEED’s five-country leadership team for approval. This is the main avenue by which SUCCEED ensures the use of data is for valid research. Once approved, those accessing the project data are required to sign a data access/confidentiality agreement form. After an 18-month embargo period, data are prepared for upload to LSHTM's Data Compass site, in line with FCDO UK’s guidance on data sharing. The prospect of data sharing is stated in participant information sheets and consent forms.

## References

[1] Whiteford HA, Ferrari AJ, Degenhardt L, et al. The global burden of mental, neurological and substance use disorders: an analysis from the Global Burden of Disease Study 2010. PLoS one. 2015;10:e0116820. 25658103 10.1371/journal.pone.0116820 PMC4320057

[2] Moreno-Küstner B, Martin C, Pastor L. Prevalence of psychotic disorders and its association with methodological issues. A systematic review and meta-analyses. PloS one. 2018;13:e0195687. 29649252 10.1371/journal.pone.0195687 PMC5896987

[3] Vos T, Abajobir AA, Abate KH, et al. Global, regional, and national incidence, prevalence, and years lived with disability for 328 diseases and injuries for 195 countries, 1990–2016: a systematic analysis for the Global Burden of Disease Study 2016. The Lancet. 2017;390:1211–59. 10.1016/S0140-6736(17)32154-2 PMC5605509 28919117

[4] Charlson FJ, Ferrari AJ, Santomauro DF, et al. Global epidemiology and burden of schizophrenia: findings from the global burden of disease study 2016. Schizophr Bull. 2018;44:1195–203. 29762765 10.1093/schbul/sby058 PMC6192504

[5] Hunt X, Bradshaw M, Vogel SL, Encalada AV, Eksteen S, Schneider M, Chunga K, Swartz L. Community Support for Persons with Disabilities in Low- and Middle-Income Countries: A Scoping Review. Int J Environ Res Public Health. 2022;19(14):8269. 10.3390/ijerph19148269. 10.3390/ijerph19148269 PMC9319493 35886121

[6] Fekadu A, Medhin G, Lund C, et al. The psychosis treatment gap and its consequences in rural Ethiopia. BMC Psychiatry. 2019;19:1–11. 31664977 10.1186/s12888-019-2281-6 PMC6819476

[7] Burns JK, Esterhuizen T. Poverty, inequality and the treated incidence of first-episode psychosis: an ecological study from South Africa. Soc Psychiatry Psychiatr Epidemiol. 2008;43:331–5. 18253683 10.1007/s00127-008-0308-2

[8] Ayano G, Tesfaw G, Shumet S. The prevalence of schizophrenia and other psychotic disorders among homeless people: a systematic review and meta-analysis. BMC Psychiatry. 2019;19:1–14. 31775786 10.1186/s12888-019-2361-7 PMC6880407

[9] Baranyi G, Scholl C, Fazel S, et al. Severe mental illness and substance use disorders in prisoners in low-income and middle-income countries: a systematic review and meta-analysis of prevalence studies. Lancet Glob Health. 2019;7:e461–71. 30879509 10.1016/S2214-109X(18)30539-4 PMC6419715

[10] Crossley NA, Alliende LM, Czepielewski LS, et al. The enduring gap in educational attainment in schizophrenia according to the past 50 years of published research: a systematic review and meta-analysis. The Lancet Psychiatry. 2022;9:565–73. 35717966 10.1016/S2215-0366(22)00121-3

[11] Kooyman I, Dean K, Harvey S, et al. Outcomes of public concern in schizophrenia. Br J Psychiatry. 2007;191:s29–36. 10.1192/bjp.191.50.s29 18019041

[12] Hinshaw SP, Stier A. Stigma as related to mental disorders. Annu Rev Clin Psychol. 2008;4:367–93. 17716044 10.1146/annurev.clinpsy.4.022007.141245

[13] World Health Organization. World mental health report: Transforming mental health for all. World Health Organization; 2022.

[14] Maiga DD, Eaton J. A survey of the mental healthcare systems in five Francophone countries in West Africa: Bénin, Burkina Faso, Côte d’Ivoire, niger and Togo. International Psychiatry. 2014;11:69–72. 31507768 10.1192/S1749367600004549 PMC6735152

[15] Lora A, Kohn R, Levav I, et al. Service availability and utilization and treatment gap for schizophrenic disorders: a survey in 50 low-and middle-income countries. Bull World Health Organ. 2012;90:47-54B. 22271964 10.2471/BLT.11.089284 PMC3260570

[16] Kohn R, Saxena S, Levav I, et al. The treatment gap in mental health care. Bull World Health Organ. 2004;82:858–66. 15640922 PMC2623050

[17] Shozi Z, Saloojee S, Mashaphu S. Experiences of coercion amongst involuntary mental health care users in KwaZulu-Natal. South Africa Frontiers in psychiatry. 2023;14:1113821. 36960456 10.3389/fpsyt.2023.1113821 PMC10027751

[18] Patel V, Chowdhary N, Rahman A, et al. Improving access to psychological treatments: lessons from developing countries. Behav Res Ther. 2011;49:523–8. 21788012 10.1016/j.brat.2011.06.012 PMC3242164

[19] Brooke-Sumner C, Selohilwe O, Mazibuko MS, et al. Process evaluation of a pilot intervention for psychosocial rehabilitation for service users with schizophrenia in North West Province. South Africa Community mental health journal. 2018;54:1089–96. 30094739 10.1007/s10597-018-0318-9

[20] Asher L, Patel V, De Silva MJ. Community-based psychosocial interventions for people with schizophrenia in low and middle-income countries: systematic review and meta-analysis. BMC Psychiatry. 2017;17:1–15. 29084529 10.1186/s12888-017-1516-7 PMC5661919

[21] Asher L, Birhane R, Weiss HA, et al. Community-based rehabilitation intervention for people with schizophrenia in Ethiopia (RISE): results of a 12-month cluster-randomised controlled trial. Lancet Glob Health. 2022;10:e530–42. 35303462 10.1016/S2214-109X(22)00027-4 PMC8938762

[22] Butura A-M, Ryan GK, Shakespeare T, et al. Community-based rehabilitation for people with psychosocial disabilities in low-and middle-income countries: a systematic review of the grey literature. Int J Ment Heal Syst. 2024;18:13. 10.1186/s13033-024-00630-0 PMC10941461 38486243

[23] Leamy M, Bird V, Le Boutillier C, et al. Conceptual framework for personal recovery in mental health: systematic review and narrative synthesis. Br J Psychiatry. 2011;199:445–52. 22130746 10.1192/bjp.bp.110.083733

[24] De Silva MJ, Breuer E, Lee L, et al. Theory of Change: a theory-driven approach to enhance the Medical Research Council’s framework for complex interventions. Trials. 2014;15:267. 10.1186/1745-6215-15-267. 2014/07/06. 24996765 10.1186/1745-6215-15-267 PMC4227087

[25] Asher L, Fekadu A, Hanlon C, et al. Development of a community-based rehabilitation intervention for people with schizophrenia in Ethiopia. PLoS ONE. 2015;10: e0143572. 26618915 10.1371/journal.pone.0143572 PMC4664267

[26] Asher L, Hanlon C, Birhane R, et al. Community-based rehabilitation intervention for people with schizophrenia in Ethiopia (RISE): a 12 month mixed methods pilot study. BMC Psychiatry. 2018;18:250. 30075715 10.1186/s12888-018-1818-4 PMC6091097

[27] De Silva MJ, Breuer E, Lee L, et al. Theory of change: a theory-driven approach to enhance the Medical Research Council’s framework for complex interventions. Trials. 2014;15:1–13. 24996765 10.1186/1745-6215-15-267 PMC4227087

[28] Abayneh S, Lempp H, Hanlon C. Participatory action research to pilot a model of mental health service user involvement in an Ethiopian rural primary healthcare setting: study protocol. Research involvement and engagement. 2020;6:1–14. 31934350 10.1186/s40900-019-0175-x PMC6951014

[29] Burgess R, Sanguineti MCD, Maldonado-Carrizosa D, et al. Using participatory action research to reimagine community mental health services in Colombia: a mixed-method study protocol. BMJ Open. 2022;12: e069329. 36549743 10.1136/bmjopen-2022-069329 PMC9772630

[30] Ryan GK, Kamuhiirwa M, Mugisha J, et al. Peer support for frequent users of inpatient mental health care in Uganda: protocol of a quasi-experimental study. BMC Psychiatry. 2019;19:374. 10.1186/s12888-019-2360-8. 31783827 10.1186/s12888-019-2360-8 PMC6883561

[31] Blickem C, Dawson S, Kirk S, et al. What is asset-based community development and how might it improve the health of people with long-term conditions? A realist synthesis Sage Open. 2018;8:2158244018787223.

[32] Moran GS, Kalha J, Mueller-Stierlin AS, et al. Peer support for people with severe mental illness versus usual care in high-, middle- and low-income countries: study protocol for a pragmatic, multicentre, randomised controlled trial (UPSIDES-RCT). Trials. 2020;21:1–15. 10.1186/s13063-020-4177-7. 32357903 10.1186/s13063-020-4177-7 PMC7195705

[33] Mpango R, Kalha J, Shamba D, et al. Challenges to peer support in low- and middle-income countries during COVID-19. Globalization & Health 2020;16:N.PAG-N.PAG. 10.1186/s12992-020-00622-y. 10.1186/s12992-020-00622-y PMC7517058 32977816

[34] Puschner B, Repper J, Mahlke C, Nixdorf R, Basangwa D, Nakku J, Ryan G, Baillie D, Shamba D, Ramesh M, Moran G. Using peer support in developing empowering mental health services (UPSIDES): background, rationale and methodology. Ann Glob Health. 2019;85(1). 10.5334/aogh.2435 PMC6634474 30951270

[35] Proctor E, Silmere H, Raghavan R, et al. Outcomes for implementation research: conceptual distinctions, measurement challenges, and research agenda. Administration and policy in mental health and mental health services research. 2011;38:65–76. 20957426 10.1007/s10488-010-0319-7 PMC3068522

[36] Hoffmann TC, Glasziou PP, Boutron I, et al. Better reporting of interventions: template for intervention description and replication (TIDieR) checklist and guide. Bmj 2014;348. 10.1136/bmj.g1687 24609605

[37] Moher D and Chan AW. SPIRIT (standard protocol items: recommendations for interventional trials). Guidelines for Reporting Health Research: a user's manual 2014:56–67.

[38] Omobowale OG, Rachel; Ryan, Grace K et al. Living with psychosis in West and Southeast Africa: SUCCEED Africa’s four-country situation analysis. 2024. In press. 10.1017/gmh.2024.122 PMC11729528 39811625

[39] World Health Organization. The ICD-10 Classification of mental and behavioural disorders. Geneva: World Health Organization; 1993.

[40] Puschner B, Repper J, Mahlke C, et al. Using peer support in developing empowering mental health services (UPSIDES): background, rationale and methodology. Ann Global Health. 2019;85. 10.5334/aogh.2435 PMC6634474 30951270

[41] Funk M, Bold ND. WHO’s QualityRights initiative: transforming services and promoting rights in mental health. Health Hum Rights. 2020;22:69. 32669790 PMC7348459

[42] Asher L, De Silva M, Hanlon C, et al. Community-based rehabilitation intervention for people with schizophrenia in Ethiopia (RISE): study protocol for a cluster randomised controlled trial. Trials. 2016;17:1–14. 27342215 10.1186/s13063-016-1427-9 PMC4919867

[43] Cohen A, Eaton J, Radtke B, et al. Three models of community mental health services in low-income countries. Int J Ment Heal Syst. 2011;5:1–10. 10.1186/1752-4458-5-3 PMC3040158 21266051

[44] World Health Organization, Consortium IDD. Community-based rehabilitation: CBR guidelines. 2010. 26290927

[45] Raja S, Underhill C, Shrestha P, et al. Integrating mental health and development: a case study of the BasicNeeds Model in Nepal. PLoS Med. 2012;9: e1001261. 22802741 10.1371/journal.pmed.1001261 PMC3393669

[46] Singla DR, Kohrt BA, Murray LK, et al. Psychological treatments for the world: lessons from low-and middle-income countries. Annu Rev Clin Psychol. 2017;13:149–81. 28482687 10.1146/annurev-clinpsy-032816-045217 PMC5506549

[47] Organization WH. The world health organization quality of life (WHOQOL)-BREF. 2004. World Health Organization.

[48] Becchi A, Rucci P, Placentino A, et al. Quality of life in patients with schizophrenia—comparison of self-report and proxy assessments. Soc Psychiatry Psychiatr Epidemiol. 2004;39:397–401. 15133597 10.1007/s00127-004-0761-5

[49] Vahedi S. World Health Organization Quality-of-Life Scale (WHOQOL-BREF): analyses of their item response theory properties based on the graded responses model. Iran J Psychiatry. 2010;5:140. 22952508 PMC3395923

[50] Kalfoss MH, Reidunsdatter RJ, Klöckner CA, et al. Validation of the WHOQOL-Bref: psychometric properties and normative data for the Norwegian general population. Health Qual Life Outcomes. 2021;19:1–12. 33413455 10.1186/s12955-020-01656-x PMC7792093

[51] Kohrt BA, Jordans MJ, Rai S, et al. Therapist competence in global mental health: development of the ENhancing Assessment of Common Therapeutic factors (ENACT) rating scale. Behav Res Ther. 2015;69:11–21. 25847276 10.1016/j.brat.2015.03.009 PMC4686771

[52] Kohrt BA, Ramaiya MK, Rai S, et al. Development of a scoring system for non-specialist ratings of clinical competence in global mental health: a qualitative process evaluation of the Enhancing Assessment of Common Therapeutic Factors (ENACT) scale. Global Mental Health. 2015;2: e23. 28593049 10.1017/gmh.2015.21 PMC5269630

[53] WHO. The World Health Organization World Mental Health Composite International Diagnostic Interview (WHO WMH-CIDI), https://www.hcp.med.harvard.edu/wmhcidi/ (2017, accessed 3 October 2022).

[54] Asher L, Birhane R, Teferra S, et al. “Like a doctor, like a brother”: achieving competence amongst lay health workers delivering community-based rehabilitation for people with schizophrenia in Ethiopia. PLoS on. 2021;16:e0246158. 10.1371/journal.pone.0246158 PMC7906313 33630893

[55] Vlădescu C, Scîntee SG, Olsavszky V et al. Romania: health system review. World Health Organization, on behalf of the European Observatory on Health. 2016. 27603897

[56] Kohler U and Kreuter F. Data analysis using Stata. Stata press, 2005.

[57] Atlas SB. ti for qualitative data analysis. Perspect Educ. 2002;20:65–75.

[58] Lacey A, Luff D. Qualitative data analysis. Trent focus Sheffield, 2001.

[59] Ritchie J, Spencer L, Bryman A, et al. Qualitative data analysis for applied policy research. Analyzing qualitative data. 1994;173:194.

[60] Acts UPG. Mental Capacity Act 2005. 2005.

[61] Jeste DV, Palmer BW, Appelbaum PS, et al. A new brief instrument for assessing decisional capacity for clinical research. Arch Gen Psychiatry. 2007;64:966–74. 17679641 10.1001/archpsyc.64.8.966

[62] Medicine LSoHaT. LSHTM Research Online. 2023.

[63] Ryan G, Iemmi V, Hanna F, et al. Mental health for sustainable development: a topic guide for development professionals. 2020.

[64] Omigbodun O, Ryan G, Fasoranti B, et al. Reprioritising global mental health: psychoses in sub-Saharan Africa. Int J Ment Heal Syst. 2023;17:1–14. 10.1186/s13033-023-00574-x PMC10043866 36978186

[65] Oliver K, Kothari A, Mays N. The dark side of coproduction: do the costs outweigh the benefits for health research? Health research policy and systems. 2019;17:1–10. 30922339 10.1186/s12961-019-0432-3 PMC6437844

[66] Lee YY, Buyanga M, Mehta A, et al. Cracks that let the light in: collective reflections on integrating lived experience of psychosis in research and policy in the context of a global commission. Comm Mental Health J. 2023;59:819–25. 10.1007/s10597-023-01118-w 36939989

