# Supplementary material for: SUCCEED Africa: protocol for a multi-method pilot study of a community-based intervention for people with psychosis in Sierra Leone, Nigeria, Zimbabwe and Malawi
Source: Pilot Feasibility Stud. 2024 Aug 27;10:114. doi: 10.1186/s40814-024-01536-x (PMC11348716; doi:10.1186/s40814-024-01536-x)
Supplement: Supplementary file 1 — Additional file 1: Cross-Site Theory of Change [file 40814_2024_1536_MOESM1_ESM.pdf]

Additional File #1: Cross-Site Theory of Change

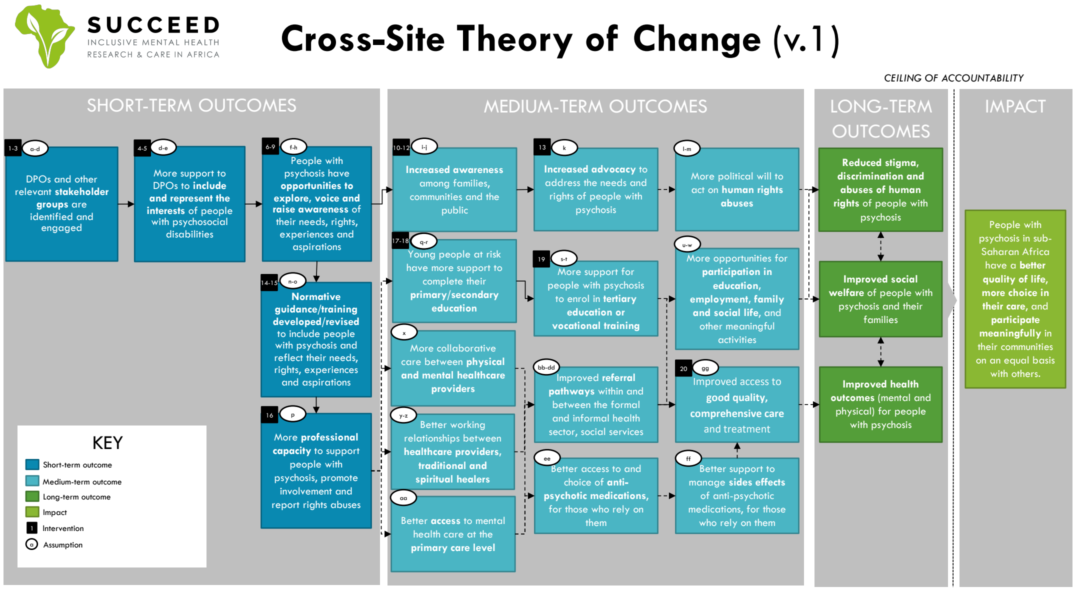

Assumptions

1. DPOs and other key stakeholders are willing and able to participate
2. Resources are sufficient to support accessible and effective meetings and communications
3. Virtual meetings and communications can provide an equally accessible and effective alternative to face-to-face meetings, where required (e.g., due to Covid-19 restrictions)
4. DPOs do not face operational challenges/threats (e.g., lack of funding) that inhibit their functioning
5. There are no barriers to people with psychosocial disability participating in DPOs
6. People with psychosocial disabilities and their families are willing and able to participate in self-help groups
7. People with psychosocial disabilities and other stakeholders are willing to collaborate effectively and share power for the purposes of co-production and co-delivery
8. People with psychosocial disabilities can safely self-disclose/participate in awareness-raising, despite stigma and discrimination, including from families
9. Families, communities, and the public at large will be receptive to messaging
10. Competing priorities in the media (e.g., Covid-19) do not drown out/dilute messaging
11. Key stakeholders will be willing and able to translate advocacy training into action, without expecting further incentives
12. Political will isn't diluted by more urgent issues (e.g., Covid-19, conflict, etc.)
13. Environment of relative political stability (election cycle doesn't interfere) and receptivity to advocacy
14. SUCCEED teams/advisors have sufficient technical knowledge and skills to adapt/produce high-quality trainings

15. SUCCEED teams are willing to collaborate effectively and share power for purposes of co-production and co-delivery
16. Professionals will be willing and able to participate in training
17. Teachers and other professionals in schools are willing to work with us to make change
18. Health/social services exist that have the capacity and willingness to receive referrals of young people with psychosis
19. Appropriate opportunities for tertiary education/vocational training exist in the area
20. SUCCEED team have or can partner with other organisations that have the skills/expertise and resources to establish livelihoods activities
21. These opportunities for participation exist, even for people without psychosocial disabilities but perhaps other disadvantages
22. SUCCEED's resources/influence are sufficient to ensure these opportunities can be made accessible to people with psychosocial disabilities
23. Improved participation yields positive experiences for people with psychosocial disabilities (does not simply expose them to new situations of adversity in difficult environments).
24. Physical/mental healthcare providers will be willing to work together and with SUCCEED
25. Traditional/spiritual healers and healthcare providers will be willing to work together and with SUCCEED
26. People with psychosocial disabilities and their families are comfortable with both biomedical and traditional/spiritual healing approaches
27. Some prior integration of mental health into primary care has already taken place
28. Barriers to access do not interrupt participants' uptake of referrals
29. Services are willing and able to handle possible increase in demand through referrals
30. Relationships between services do not deteriorate
31. It is possible to make drugs available (especially second-line drugs) at affordable prices
32. It is possible to effectively manage side effects, with the resources available (human resources, drugs, special accommodations, e.g., at work/home)
33. Factors beyond our control do not negatively impact quality (e.g., hospital strikes)
